# Higher-Order Synaptic Interactions Coordinate Dynamics in Recurrent Networks

**DOI:** 10.1371/journal.pcbi.1005078

**Published:** 2016-08-19

**Authors:** Brendan Chambers, Jason N. MacLean

**Affiliations:** 1 Committee on Computational Neuroscience, University of Chicago, Chicago, Illinois, United States of America; 2 Department of Neurobiology, University of Chicago, Chicago, Illinois, United States of America; Frankfurt Institute for Advanced Studies, GERMANY

## Abstract

Linking synaptic connectivity to dynamics is key to understanding information processing in neocortex. Circuit dynamics emerge from complex interactions of interconnected neurons, necessitating that links between connectivity and dynamics be evaluated at the network level. Here we map propagating activity in large neuronal ensembles from mouse neocortex and compare it to a recurrent network model, where connectivity can be precisely measured and manipulated. We find that a dynamical feature dominates statistical descriptions of propagating activity for both neocortex and the model: convergent clusters comprised of fan-in triangle motifs, where two input neurons are themselves connected. Fan-in triangles coordinate the timing of presynaptic inputs during ongoing activity to effectively generate postsynaptic spiking. As a result, paradoxically, fan-in triangles dominate the statistics of spike propagation even in randomly connected recurrent networks. Interplay between higher-order synaptic connectivity and the integrative properties of neurons constrains the structure of network dynamics and shapes the routing of information in neocortex.

## Author Summary

Active networks of neurons exhibit beyond-pairwise dynamical features. In this work, we identify a canonical higher-order correlation in network dynamics and trace its emergence to synaptic integration. We find that temporally coordinated firing preferentially occurs at sites of fan-in triangles—a synaptic motif which coordinates presynaptic timing, leading to greater likelihood of postsynaptic spiking. The influence of fan-in clustering leads to the surprising emergence of non-random routing of spiking in random synaptic networks. When synaptic weights are made artificially stronger in simulation, so that cooperative input is less crucial, dynamics are no longer dominated by fan-in triangles but instead more closely reflect the random synaptic network. Thus, the emergence of fan-in clustering in maps of synaptic recruitment is a collective property of individually weak connections in neuronal networks. Because higher-order interactions are necessary to shape the timing of presynaptic inputs, activity does not propagate uniformly through the synaptic network. Like water finding the deepest channels as it flows downhill, spiking activity follows the path of least resistance and is routed through triplet motifs of connectivity. These results argue that clustered fan-in triangles are a canonical network motif and mechanism for spike routing in local neocortical circuitry.

## Introduction

Understanding any complex system requires a mechanistic account of how dynamics arise from underlying architecture. Patterns of connections shape dynamics in diverse settings ranging from electric power grids to gene transcription networks[1–5]. It is critical to establish how synaptic connectivity orchestrates the dynamics of propagating activity in neocortical circuitry, since dynamics are closely tied to cortical computation. For example, trial-to-trial differences in network dynamics[6–9] can be used to decode sensory inputs and behavioral choice[10,11]. It is particularly important to understand the transformation from connectivity to activity within local populations of neurons since this is the scale at which the majority of connections arise. Locally, neocortical neurons are highly interconnected, and their connectivity schemes are characterized by the prevalence of specific motifs[12]. At the level of local populations, functional coordination has been demonstrated in diverse ways, *e*.*g*. on the basis of active neurons[13,14] and their correlation patterns[15]. Yet predicting population responses on the basis of pairwise connections alone has proven to be difficult.

Establishing a mechanistic link between connectivity and dynamics in neocortical networks is intricate and non-trivial because individual neurons themselves are complex computational units[16–20]. Fundamentally, neurons are state dependent non-linear integrators of synaptic input[21–23]. When neurons in neocortex process information, they are generally subjected to numerous synaptic inputs which activate diverse receptors, and concomitant gating of voltage-dependent channels[24–26]. In consequence, neocortical neurons tend to operate in a high-conductance state, which lessens the impact of any one synaptic input[21,27]. Because inputs are weak individually, collective synaptic bombardments are necessary to depolarize a neuron to threshold for action potential generation. As a result, it is difficult to predict the flow of activity through a synaptic network based solely on knowledge of single connections, without the context of ongoing activity in the entirety of the system.

Network models are an important tool for linking synaptic connectivity to dynamics in neocortex because they enable precise measurement and manipulation of simulated connectivity. In this work, we generate networks comprised of leaky integrate-and-fire model neurons with naturalistic dynamics that mimic recordings from superficial neocortical layers. Despite random synaptic topology in the model network, we find that small-world topological organization emerges in maps of propagating activity. This paradoxical divergence of dynamics from synaptic connectivity is not explained by coactivity alone. Rather, recruitment preferentially occurs in a selective subset of active connected pairs.

In the model, activity is preferentially routed through clustered fan-in triangles, despite their statistical scarcity. Because they result in coordinated presynaptic timing, fan-in triangle motifs are particularly effective for spike generation. By comparison, among neurons converging on a common target but lacking presynaptic interconnectivity, presynaptic timing is less synchronous on average, and postsynaptic recruitment is less likely. Moreover, when we decrease the need for cooperative presynaptic action, by doubling synaptic weights in network models, the fan-in triangle motif becomes significantly less prevalent. We evaluate the prediction of our model using high speed two-photon imaging of emergent network activity *ex vivo*, in somatosensory cortex. We verify that propagating activity in real neuronal networks has small-world characteristics and elevated clustering, Decomposing this clustering, we discover that neocortical circuitry also manifests propagating activity that is dominated by the fan-in triangle motif. These results suggest a mechanistic account for the widespread findings of clustered activity in neuronal populations [14,28–31]. We suggest that clustered fan-in triangles are a canonical building block for reliable cortical dynamics.

## Results

### Representing activity and connectivity with directed graphs

**Fig 1:**
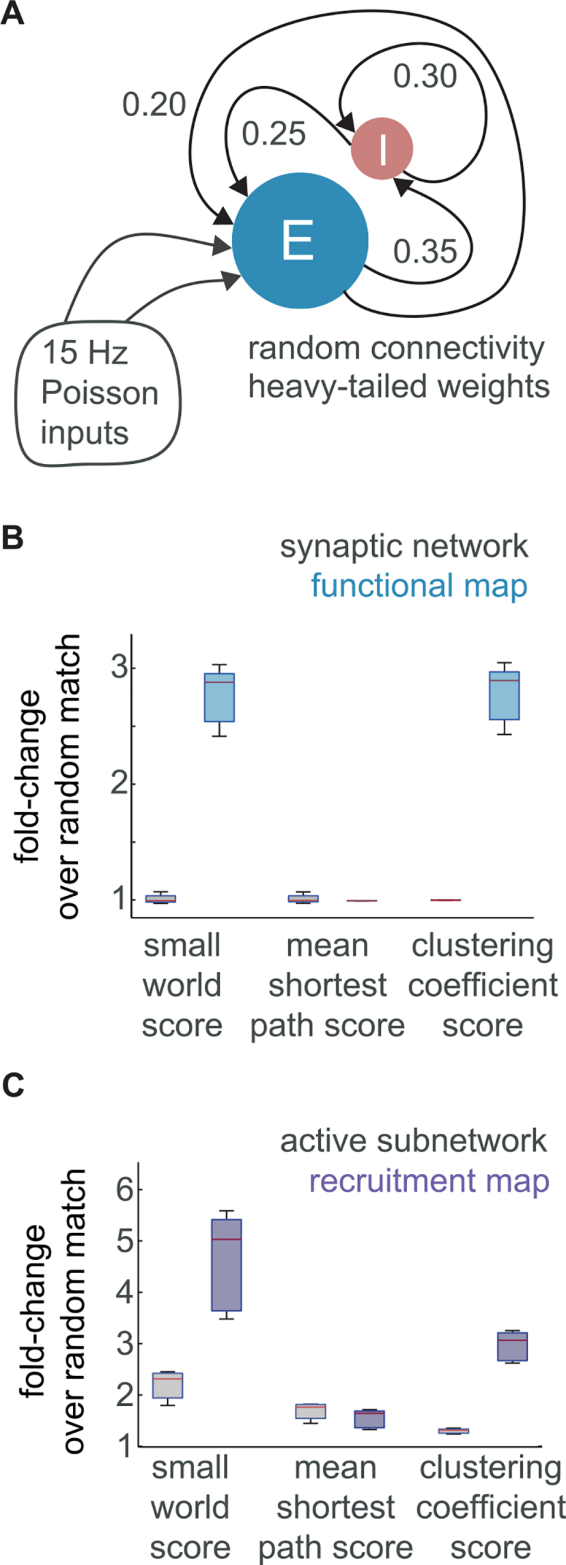
Emergent functional networks are structured despite random synaptic connectivity. (a) Integrate-and-fire neurons with conductance-based synapses were connected randomly according to source and target class (200 inhibitory and 1000 excitatory cells). Activity was initiated with 50 ms of independent Poisson inputs. (b) Box plots of the fold change over random for the small world score, shortest path length score, and clustering coefficient score in the synaptic network and the functional network. (c) Box plots of the fold change over random for the small world score, shortest path length score, and clustering coefficient score in the active subnetwork and the recruitment network.

Multineuronal dynamics are the computational substrate for sensation and behavior, implemented by synaptic architectures. Propagating multineuronal activity arises from three main sources: the underlying connectivity itself, recent network history, and the non-linear integrative properties of individual neurons. Here, multineuronal activity was modeled using conductance-based leaky integrate-and-fire neurons, stimulated with brief periods of Poisson input and recorded during self-sustained firing (Fig 1A). Model neurons were connected with heterogeneous synaptic weights drawn from a heavy-tailed distribution, in a random arrangement (Erdős-Rényi; *p*_*ee*_ = 0.2). Simulated dynamics were asynchronous, irregular, and sparse, with critical branching (see Methods).

**Fig 2:**
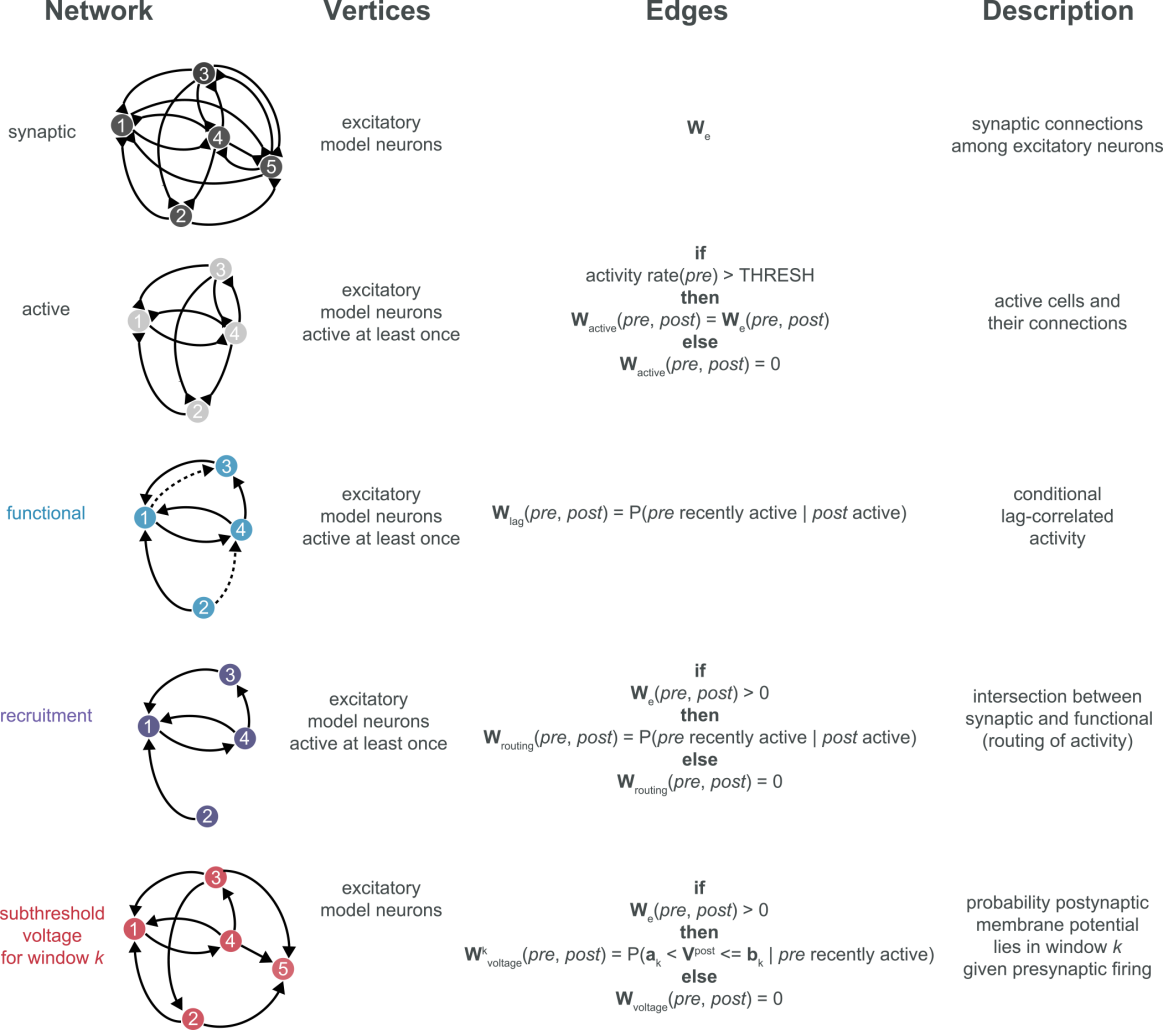
Glossary of network definitions.

A synaptic network was constructed for each simulation, consisting of excitatory model neurons and their synaptic connectivity. For each structural iteration of the model we generated three distinct maps of activity (and in two of the cases, multiplex connectivity and activity): a *functional network*, the *active subnetwork*, and a *recruitment network* (Fig 2). Edges in the functional network summarized network dynamics and represented frequency of lagged firing between every pair of nodes (with maximum interspike interval *T* = 25 ms; see Methods). The active subnetwork was a subgraph of the synaptic network and consisted of model neurons active at least once and all their interconnections (regardless of lagged firing relationships). Finally, the recruitment network was a subgraph of the functional network defined by its intersection with the synaptic network, to map the routing of activity through synaptic interactions. In this way, non-zero edges in the recruitment network linked synaptically connected nodes that also spiked sequentially in the interval *T* at least once. For *T* = 25 ms, 10.9 ± 3.52 excitatory presynaptic input spikes immediately preceded each postsynaptic spike (mean±std).

Surprisingly, although underlying synaptic connectivity was Erdős-Rényi (i.e. random), functional activity networks were small world (Fig 1B)[32]. To judge the small world character of these networks, global clustering coefficient and characteristic path were normalized by their respective abundances in density-matched Erdős-Rényi networks and combined as a quotient[33]. Comparison with density-matches was important given that sparseness itself results in enhanced smallworldness[34].

Functional networks were marked by significantly increased small world scores (functional network: 2.8±0.23; synaptic network: 1.0±0.035; *n* = 5, *p* = 0.0079, *Wilcoxon rank-sum*) resulting from increased clustering (function: 2.8±0.23; synaptic network: 1.0±0.035, *n* = 5, *p* = 0.0079), with characteristic path lengths similar to random-matches (function: 1.0±6.4*x*10^-4^; synaptic network: 0.99±0.033; *n* = 5, *p* = 0.69). The lag interval *T* was chosen to encompass important network timescales for synaptic plasticity and integration[35,36]. We also generated functional networks using intervals of 10 and 50 ms, which showed that the emergence of non-random features does not depend strongly on choice of *T* (functional network for *T* = 10ms: small world ratio 3.2±0.24, *n* = 5, *p* = 0.0079; functional network for *T* = 50ms: small word ratio 2.6±0.22, *n* = 5, *p* = 0.0079).

Given modest sampling conditions (e.g. binning near timescales of synaptic integration), functional relationships can indicate locations of probable synaptic recruitment[35]. However, a subset of edges in functional networks are 'false positives'—they reflect polysynaptic relationships and other combined statistical dependencies rather than monosynaptic connectivity and recruitment[35,37]. To determine whether these measurement artifacts were responsible for the statistical differences between functional and synaptic networks, we turned to recruitment networks. Pruned of false positives, recruitment networks were significantly more small world than functional networks constructed from the same activity (4.6±0.87; *n* = 5, *p* = 0.0079), with even shorter characteristic paths (recruitment: 0.65±0.072, *n* = 5, *p* = 0.0079 compared to function, *Wilcoxon rank-sum*) and a similar elevation in clustering (recruitment: 3.0±0.26; *n* = 5, *p* = 0.22). Thus, emergent statistical structure in the functional networks reflected coordinated timing among multiple synaptically connected neurons.

### Preferential routes for propagating activity

As demonstrated by non-random recruitment, i.e. clustering in the recruitment network, activity did not propagate homogeneously through the random topology. However, it remained a possibility that the seemingly non-random routing of activity was simply the byproduct of shared activity, without being selective on the basis of connectivity. As a control, the active subnetwork establishes the role of interactions among neurons with elevated firing rates (including pairs of neurons which never recruited one another within the interval *T*). Compared to functional networks, the corresponding active subnetwork exhibited reduced small world ratio (active network: 2.2±0.26, *n* = 5, *p* = 0.0159) and reduced clustering (1.3±0.041, *p* = 0.0079), despite somewhat shorter characteristic paths (0.60±0.055, *n* = 5, *p* = 0.0079).

If directed connections that never fired sequentially were pruned from the active subnetwork, it would attain the same binary topology as the recruitment network. Comparing the active network with the recruitment network, global clustering ratio was significantly increased (from 1.3±0.041 to 3.0±0.26, *n* = 5, *p* = 0.0079, *Wilcoxon rank-sum*). Thus, the select connections which were directly involved in propagation of spiking activity were more clustered than activated connections as a whole (Fig 1C).

We next evaluated whether neuronal pairs that never fired sequentially differed from those that did. Comparisons were performed between in-degree matched samples. Connected neurons that never fired in succession shared significantly fewer neighbors than those that did fire sequentially at least once (*n* = 500 pairs, *p* = 3.1 *x* 10^−17^, *Wilcoxon rank-sum*). In the model, activity was selectively routed through interconnected neighborhoods.

### Deconstructing patterns of directed clustering

**Fig 3:**
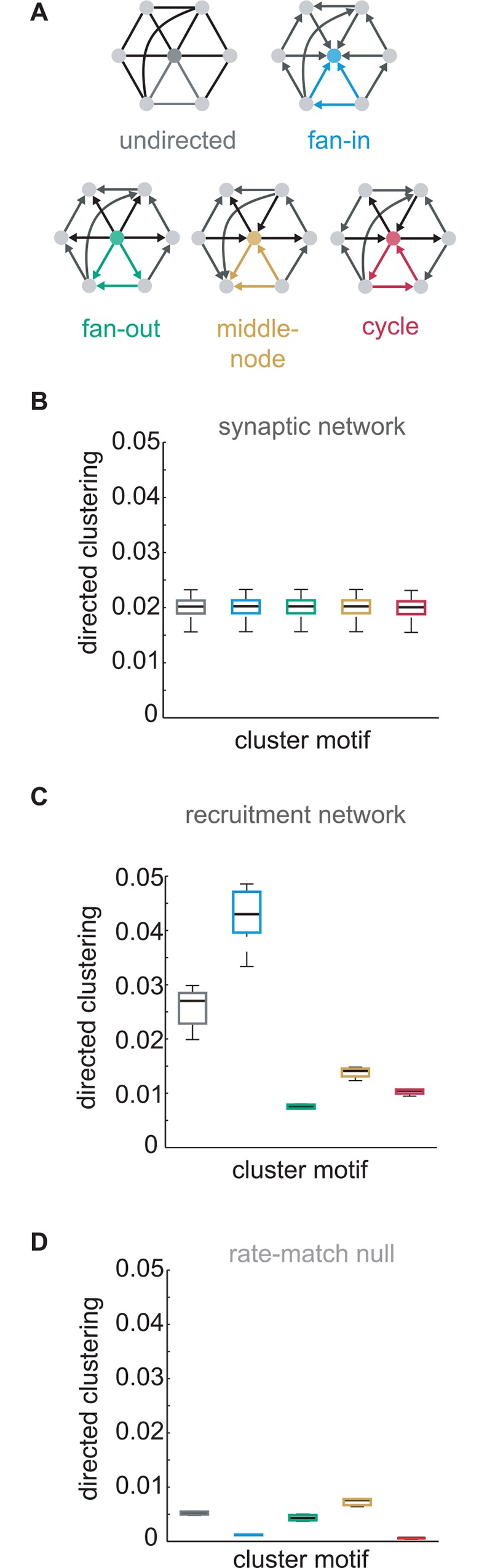
Clustered fan-in triangle motifs dominate recruitment networks. (a) Scheme for factoring transitive clustering into constituent directed patterns. (b) Boxplots of the prevalence of the directed patterns in the randomly connected synaptic network. (c) Boxplots of the prevalence of the directed patterns in the recruitment network. (d) Boxplots of the prevalence of directed patterns in nulls generated from rate-matched Poisson populations without synaptic interactions analyzed with iterative Bayesian inference.

Connectivity within a triplet is the simplest way two nodes can share a common neighbor and be clustered. However, this measure fails to account for the direction of connection. Since direction is crucial in synaptic communication, we turned to a formulation which differentiates directed triangle motifs[38]. From the perspective of a reference postsynaptic neuron, clustered neighbors can be arranged into four kinds of three-edge triangle motifs: fan-in, fan-out, middleman, and cycle arrangements (Fig 3A). Taken in isolation, fan-in, middle-node, and cycle triangles are isomorphic to one another through rotation, i.e. dependent on labeling the reference node (which is necessary to compute local clustering). Measures of undirected clustering can be decomposed fractionally into these four components. Because the underlying model synaptic connectivity was random, none of the four triangle motifs were more prevalent than the others, and each contributed equally to synaptic clustering (Fig 3B). By contrast, in recruitment networks, fan-in triangle motifs were highly overrepresented (Fig 3C). The overrepresentation of fan-in triangle motif was also present in the functional network: for example, iterative Bayesian inference[35] was sensitive to asymmetric directed clustering in model activity (fan-in: 0.38±0.052, fan-out: 0.29±0.032, middleman: 0.19±0.016, cycle: 0.15±0.0076; mean±std, threshold at the 95^th^ percentile).

**Fig 4:**
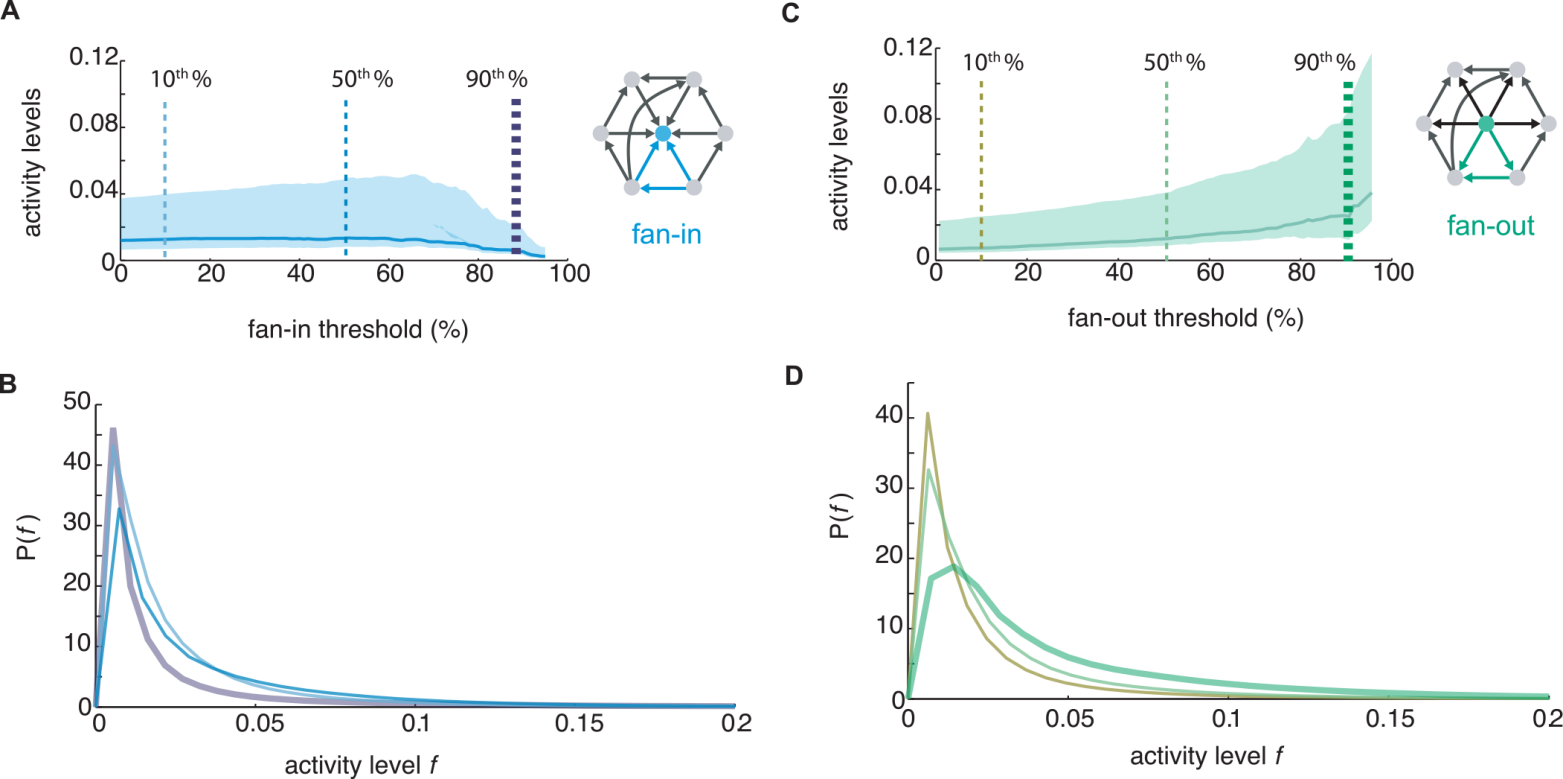
Fan-in triangle motifs are not simply the result of firing rate. (a) Median firing rate across all fan-out clustering thresholds (middle quartiles shaded). (b) Probability distribution of firing rates for fan out triangle motif. Firing rate distributions for subpopulations thresholded to exclude the bottom 10% (mustard), 50% (light green), and 90% (dark green) of fan-out clustered model neurons. *(c)* median firing rate across all fan-in clustering thresholds (middle quartiles shaded). (d) Probability distribution of firing rates for fan in triangle motif. Reference cells with high fan-in clustering had lower firing rates than the population as a whole: bottom10% (light blue), 50% (blue), and 90% (purple) of fan-in clustered model neurons.

To understand whether these higher order asymmetric features emerge from chance correlations tied to firing rates, we generated Poisson populations that were rate-matched on a neuron-by-neuron and trial-by-trial basis. This resulted in an inhomogeneous distribution of firing rates across all trails. Our Poisson null populations had identical expected spike counts as model activity in each 100ms bin but no synaptic interactions and no causal propagation of activity. Undirected clustering was significantly lower in iterative Bayesian maps of uncoupled Poisson rate-matched activity compared to connected network models (Poisson rate-match: 0.0052±3.6*x*10^-4^; simulated activity: 0.024±0.013; *Wilcoxon rank-sum p* = 0.036; *n* = 3), and the fan-in triangle motif was not elevated relative to other clustering patterns (Fig 3D). The Poisson populations demonstrated that elevated fan-in triangle motifs do not result trivially from the analysis procedure but instead are the result of synaptic interactions between neurons. Interestingly, we found that model neurons with high fan-out clustering were characterized by elevated firing rates (Fig 4A and 4B), but model neurons which comprised the fan-in triangle motif actually contracted towards low firing rates (Fig 4C and 4D). Fan-in triangles were more abundant in propagating activity than would be expected from their frequency in the synaptic network or component firing rates alone.

Like undirected clustering, the emergence of fan-in clustering in maps of propagating activity was robust to choice of *T*. Fan-in clustering was highly elevated in recruitment maps for *T* = 10 ms (undirected: 0.0068±0.0007; fan-in 0.011±0.0017; fan-out: 0.0028±0.0001; middle-node: 0.0068±0.0007; cycle: 0.0052±0.0004; mean±std for 5 simulations) and *T* = 50 ms (undirected: 0.019±0.0015; fan-in 0.027±0.0027; fan-out: 0.0077±0.0003; middle-node: 0.019±0.0013; cycle: 0.015±0.0007; mean±std for 5 simulations). Because of the different levels of sparseness in the numbers of connections these values should not be compared across values of T. Instead these analyses demonstrate that the over-representation of fan-in triangles is robust across a number of timescales.

### Activity at fan-in triangle motifs is temporally organized

**Fig 5:**
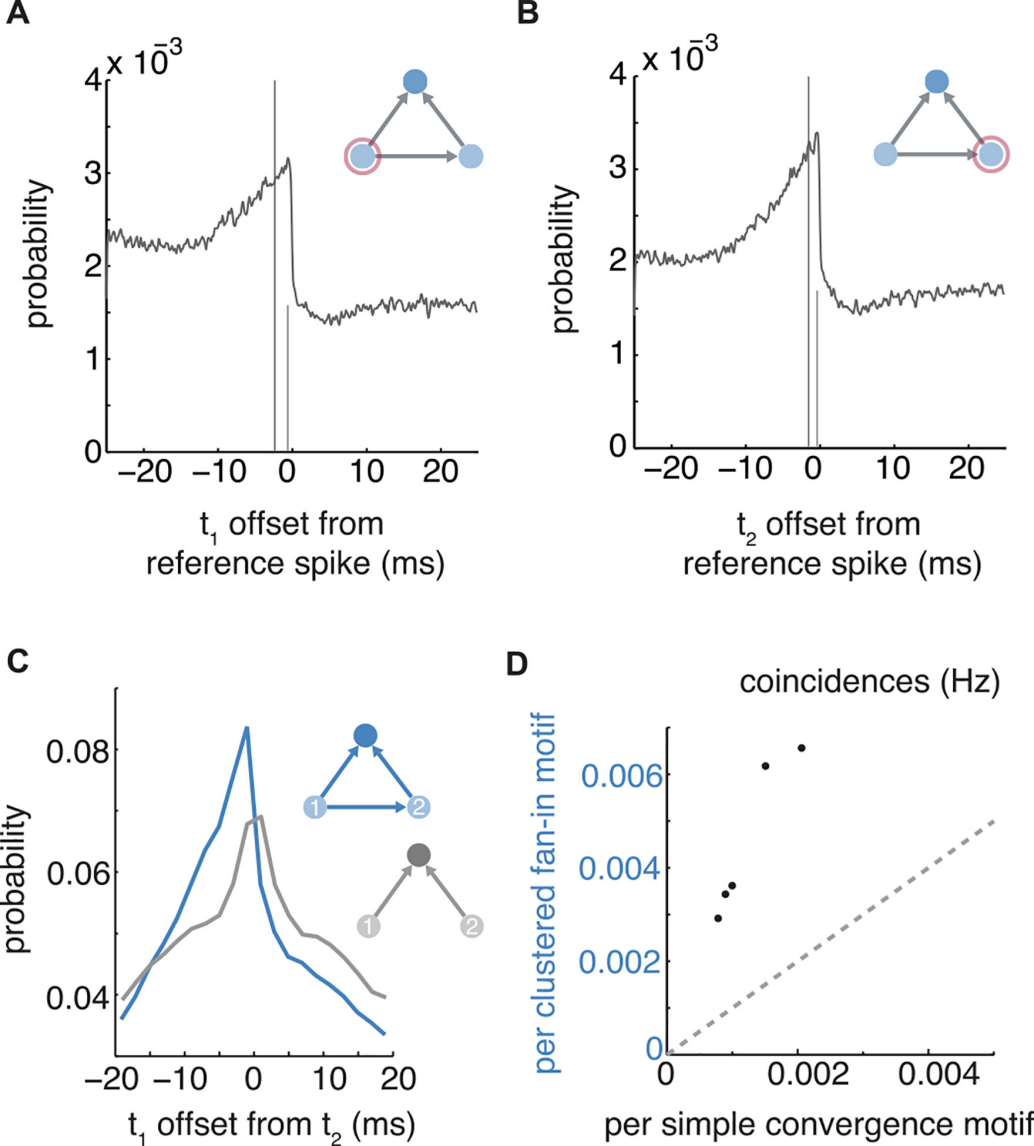
Coordinated timing among model neurons in fan-in triangle motifs. (a,b) Probablity distribution of spiking within fan-in triangle motifs. Postsynaptic spiking at *t* = 0 (tall mark, center of mass; short mark, peak). (c) Probability versus difference in presynaptic timing *t*_2_ –*t*_1_ during coincident epochs. (d) Rate of observing coincident firing (50 ms) in fan-in triangle motifs versus simple convergence.

To investigate the mechanism for overrepresentation of fan-in triangles in recruitment networks, we measured spike timing at their locations. The signature of fan-in triangle motifs is convergence from interconnected presynaptic neurons, a motif that could potentially facilitate cooperative summation of synaptic inputs. Consistent with this postulate, presynaptic neurons in fan-in triangle motifs were marked by increased probability of firing in the 10 ms prior to postsynaptic spiking (Fig 5A and 5B).

We next compared differences in presynaptic timing relationships at loci of fan-in triangle motifs compared to loci of simple convergence, to assess the role of presynaptic interconnectivity. For this analysis, random samples were obtained from epochs of *coincident firing*: 50 ms windows where every neuron in a triplet was active, centered on a spike in the postsynaptic reference neuron. To avoid confounds from juxtaposing multiple motifs, neuron triplets with any additional connections, including recurrent loops, were excluded for this specific analysis alone. As a result only fan-in triangles with exactly three interconnections were analyzed in this case. We found fan-in presynaptic neurons were stereotypically ordered in a manner consistent with the direction of their interconnection, resulting in an asymmetric distribution of intervals between their firing (Fig 5C). In addition to the temporal structure imposed by this asymmetry, mean absolute timing difference between presynaptic neurons in clustered fan-in motifs was modestly but significantly more temporally precise than were neurons in simple convergence motifs (13.5±10.2 ms compared to 14.9±10.7 ms; *Wilcoxon rank-sum* on mean-absolute timing difference, *p* = 0.0035, *n* = 1000 samples).

Moreover, we found that coincidence in fan-in triangle motifs occurred nearly twice as frequently as in motifs of simple convergence (1.9 ± 0.17 times more frequent, *mean* ± *std*; *Wilcoxon rank-sum*, *p* = 0.0079, *n* = 5 model datasets). Accounting for expected frequency of the two connection patterns in the underlying synaptic network, coincident activity is far more common at sites of fan-in triangles than at sites of simple convergence (linear regression: slope 3.0, y-intercept 0.00075, *n* = 5 simulations, *r*^2^ = 0.91, *p* = 0.011) (Fig 5D).

### Increasing clustering among active inputs with depolarization

**Fig 6:**
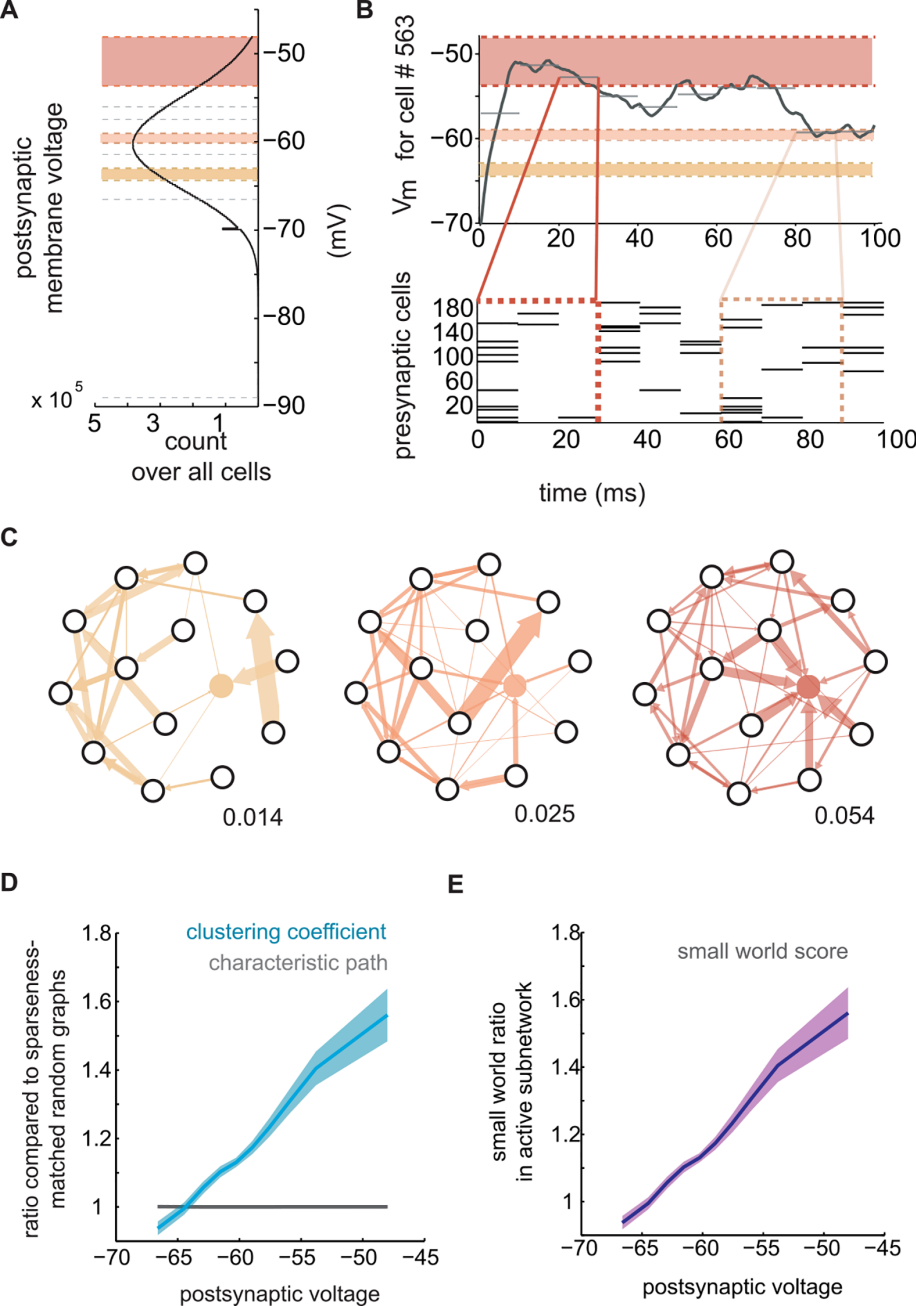
The prevalence of fan-in triangle motifs increased with post-synaptic voltage. (a) Distribution of postsynaptic voltage. Shading corresponds to (b) and (c) and contain the same number of samples per voltage bin. (b) Postsynaptic voltage was mapped in relation to presynaptic spiking. (c) One example weighted directed topology for each division of the voltage distribution. (d) Ratio versus voltage (clustering coefficient: blue, characteristic path: gray, shading reflects one standard deviation). (e) Small world ratio versus voltage (shading reflects one standard deviation).

We postulated that clustering is efficacious for synaptic integration and examined whether the prevalence of clustering was predictive of postsynaptic membrane potentials. Pooling over all neurons and time bins, we binned the distribution of membrane voltages into segments that contained equal numbers of samples (Fig 6A). On average, because the model was active in the analyzed simulations, membrane voltages were depolarized from the resting equilibrium potential of -65 mV (median: -60.2 mV; lower quartile: -63.6 mV; upper quartile: -56.8 mV). To test our hypothesis, we generated functional networks that related recent presynaptic activity (within a 25 ms interval) to postsynaptic voltage (Fig 6B; see Methods*)*, yielding one network for each division of the voltage distribution (Fig 6C). These networks can be viewed as reverse correlograms conditioned on postsynaptic voltage, and differed in the statistics of their topologies across different voltage regimes. At more negative membrane potentials, the active neurons which connected to the postsynaptic reference neuron (and accounted for its recent excitatory synaptic drive) were only modestly more clustered than random sparseness-matched controls. As the postsynaptic neuron depolarized, the presynaptic nodes driving that depolarization became increasingly clustered, peaking at the threshold for firing (Fig 6D). Characteristic paths were similar to random graphs at all subthreshold voltages. As a result of elevated clustering during membrane depolarization, small world ratios peaked at the most depolarized voltages corresponding to threshold for action potential generation. These data support the hypothesis that activity among clustered presynaptic neurons is particularly effective for recruiting the postsynaptic neuron to spike.

### Emergence of higher-order features depends on mean synaptic weight

**Fig 7:**
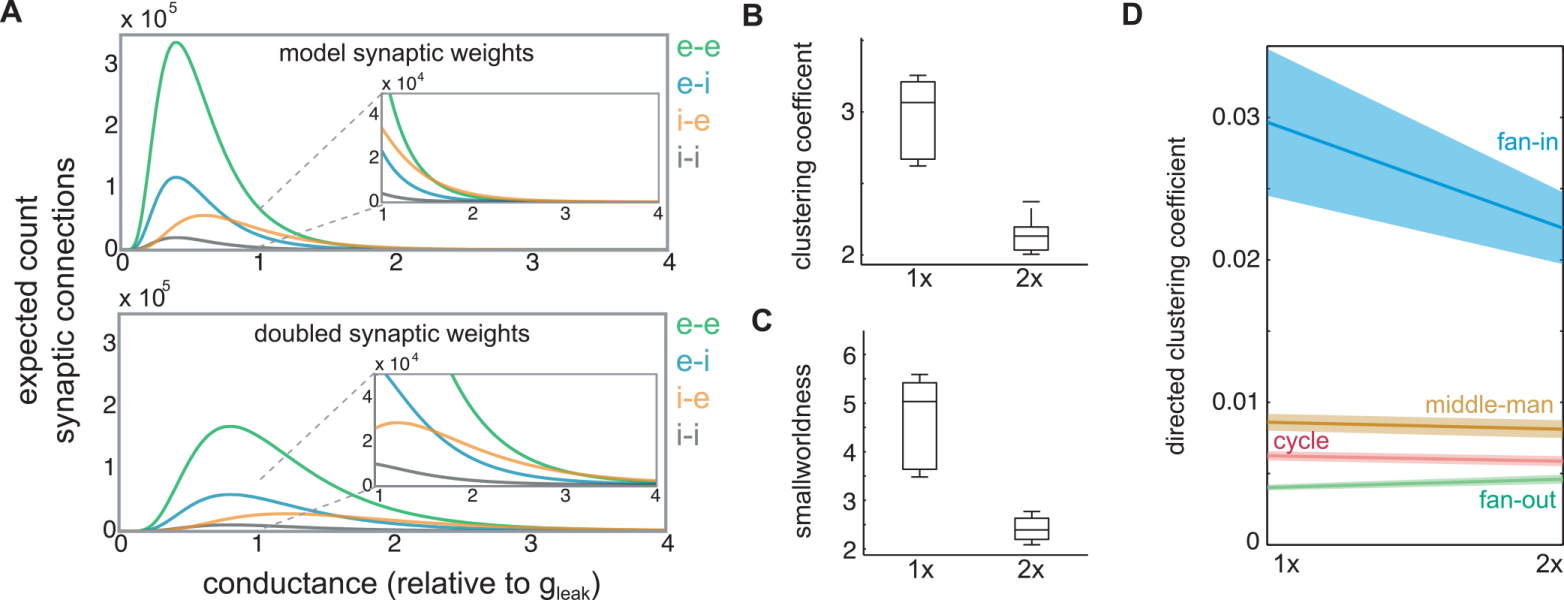
Increased synaptic weights reduced higher-order functional coordination. (a) Distribution of model synaptic weights (excitatory-excitatory: green, excitatory-inhibitory: blue, inhibitory-excitatory: orange, inhibitory-inhibitory: gray). Top: naturalistic model. Bottom: double-strength model. Inset in both cases shows zoom to better illustrate heavy tail. (b) Box plot of clustering coefficient in the two models. 2X indicated double-strength synaptic connections. (c) Box plot of small worldness in the two models. 2X indicated double-strength synaptic connections. (d) Comparison of mean directed clustering with each model iteration on either side. Each class of directed clustering is labeled in the plot.

The statistical incongruence of function and synaptic connectivity indicates that spiking activity does not flow in an egalitarian fashion through the synaptic network. Instead, patterns of local clustering influence and direct where propagating activity occurs most frequently. That is, patterns of activity are shaped by higher-order patterns in synaptic connectivity and not just pairwise couplings. To further explore the dependence of activity flow on higher order synaptic connections we evaluated postsynaptic recruitment in a network model with a modest increase in mean synaptic strength. Synaptic connections were twice as strong on average compared to the network models used throughout the remainder of this study but remained too weak to drive spiking alone (Fig 7A). The two network designs did not differ in connection density. After synaptic weights were doubled, functional networks became more similar in topology to synaptic networks (small world ratio decreased; Wilcoxon rank-sum, *p* = 0.0079, *n* = 5) (Fig 7B). The double-strength models were less clustered (Fig 7C) (Wilcoxon rank-sum, *p* = 0.0079, *n* = 5), and exhibited longer average path lengths (Wilcoxon rank-sum, *p* = 0.0079, *n* = 5). Directed clustering was compared across the two families of models. Recruitment networks were analyzed with binary edges to control for their distinct mean synaptic weights. In addition to their decreased overall clustering, the fan-in triangle motif was significantly rarer in double-strength recruitment networks (Fig 7D) (from 0.030±0.0051 to 0.022±0.0025, p = 0.030, n = 6), while the fan-out triangle motif showed a small but significant increase in abundance (from 0.0040±2.0*x*10^-4^ to 0.0046±3.2*x*10^-4^, p = 0.0043, n = 6). Stronger presynaptic inputs reduced the need for extensive postsynaptic integration, allowing individual presynaptic cells to have a more independent impact on their postsynaptic partners. As a result, statistics of propagating activity were more faithful to underlying pairwise connections in the models with increased synaptic strength.

### Fan-in triangles characterize neocortical circuit dynamics

**Fig 8:**
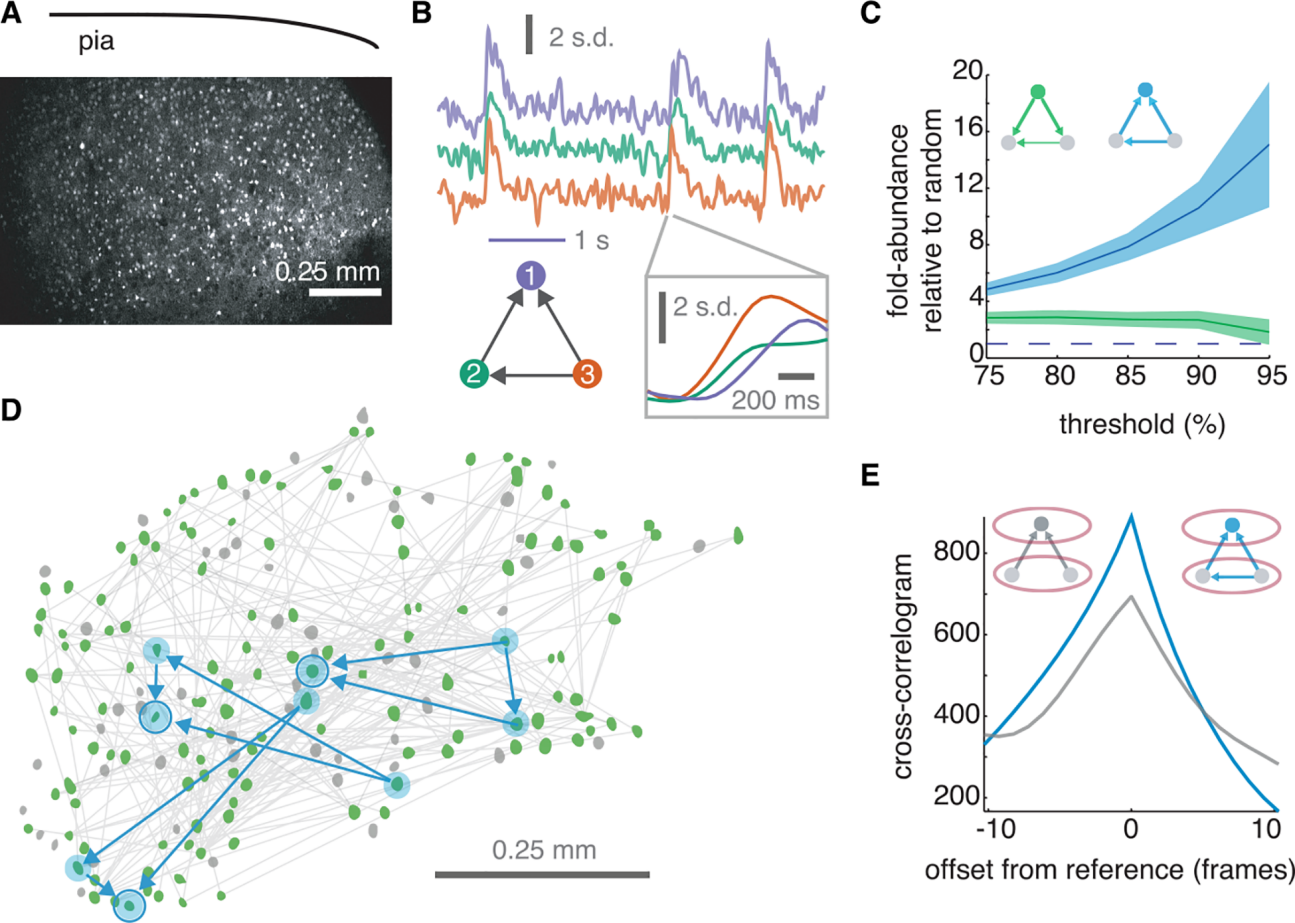
Clustering in experimentally recorded emergent cortical circuit activity was characterized by the fan-in triangle motif. (a) Two photon image of Ca2+ indicator dye in a slice of mouse somatosensory cortex. (b) Example *z*-scored fluorescent traces among functionally related neurons identified as members in a fan-in triangle motif. (c) Relative abundance of both fan-in (blue) and fan-out (green) clustering relative to density-matched random graphs as a function of inclusion threshold on inferred connections. Shading reflects one standard deviation based on bootstrap resampling for a 30% false positives rate (*n* = 100). (d) Top 5% of functional edges (light grey) and corresponding neurons (green) collected from the same field of view as a. Grey neurons were also active but were not connected with edges exceeding the cut-off. Three example fan-in triangles are illustrated with directed edges (blue arrows) and blue shading (motif specific neurons). Circle indicates reference neuron in each triangle. (e) Separate functional analysis of triplet motifs using cross-correlation. For each triplet, the product of the *z*-scored presynaptic traces were compared to the postsynaptic trace. Fan-in triangle motifs were characterized by higher levels of coordination than motifs of simple convergence.

In model simulations, fan-in triangle motifs were abundant in maps of function and recruitment. We next evaluated whether the preponderance of fan-in triangle motifs would be robust to additional complexity in single-neurons and their connections. Unlike the simple model neurons that we used for simulation, real neurons are complex elements[16] and the connections between them are structured[12,39]. If clustered fan-in triangle motifs are a general feature of high-conductance nodes in a complex system, where coordinated inputs drive integration, the fan-in triangle will be overabundant in experimental dynamics. This postulate would be falsified if all directed clustering motifs were equally common in functional networks. To investigate, we analyzed high speed imaging data (20 Hz) of spontaneous circuit activity collected *ex vivo* in mouse somatosensory cortex (Fig 8A) (following [40]). We generated functional networks from the imaged experimental data using an iterative Bayesian approach which is robust to relatively small numbers of observations [33]. We then measured the prevalence of fan-in motifs in the functional topology (Fig 8B). Importantly, iterative Bayesian inference was not biased toward detection of fan-in triangle motifs, as demonstrated with rate-matched Poisson spiking (see Fig 3D).

Though imperfect indicators, functional weights probabilistically identify the likelihood of true monosynaptic excitatory connectivity[35]. As a result, expected error rate for inferred connections can be adjusted with a sliding threshold on functional weight. Stricter thresholds yield a more accurate approximation of the underlying recruitment network at the cost of restricted sampling. Using inferred recruitment networks, beginning at the top quartile of inferred weights, directed clustering was computed in five-percentile increments. Confidence intervals were obtained using bootstrap resampling under the assumption of a 30% false-positive rate. As confidence of synaptic connectivity increased, the fan-in triangle motif became increasingly abundant and fan-out triangles less so (Fig 8C). Differences between the two motifs were significant (threshold at 95^th^ percentile, *p* = 4.8x10^-34^, n = 100 bootstrap resampled functional networks, *Wilcoxon ranksum*).

We next measured whether strong functionally coupled neurons were more spatially proximal than random pairs. We defined strong functional connections as those exceeding a 95% threshold on non-zero weights since previous work has indicated that these particular functional connections are more likely to reflect a causal synaptic connection[35]. We found that the median pairwise distance separating strong functionally connected cells was 249 μm, whereas randomly chosen pairs of neurons were separated by a median 263 μm (Wilcoxon-ranksum p = 0.0336, n^functional^ = 638, n^random^ = 10000). We then measured triplets of neurons with functional connections that form triangles to determine whether these neurons were more spatially proximal to one another than randomly chosen triplets of neurons. To investigate, proximity was quantified as the perimeter around the triangle formed by vertices at the spatial location of each neuron. Neurons in functional triangles with mutual connectivity and at least three functional connections were inscribed by perimeters of median length 807 μm, compared to median perimeter of 823 μm for randomly selected triplets that were unconstrained by direction and number of edges (Wilcoxon rank-sum p = 0.0097, n^triangles^ = 2556, n^random^ = 10,000). Interestingly, triplets of neurons connected into arrangements of either simple divergence or simple convergence (i.e. neurons in wedges, lacking interconnectedness between the common neighbors), were even more distant, inscribed by a perimeter of median 839 μm (Wilcoxon rank-sum, n^triangles^ = 2556, n^wedges^ = 14,882). Thus, clustered triplets (triangles) tended to be arranged significantly more locally than simple convergent or simple divergent triplets (wedges).

We then compared measures of clustering between the model, which was comprised of random connections, and the experimental data which almost certainly contained structured connectivity [12, 39] to evaluate how the measure of fan in and fan-out triangles depend on the underlying structural topology. To do so we used a measure of clustering propensity[41] which allowed us to make comparisons of networks which have very different connection densities. Clustering propensity (1-ΔC^fan-in^ and 1-ΔC^fan-out^) results in a normalized value where 1 is extreme clustering as seen in lattices, and 0 indicates no clustering above that expected in Erdős-Rényi random networks. For the model, fan-in clustering was scored at 0.18 ± 0.019; and for the experimental data, fan-in clustering was scored at 0.20 ± 2.0x10^-4^ (Wilcoxon ranksum *p* = 1.74x10^-4^, n^model^ = 5 simulations; n^data^ = 100 bootstrap samples). Thus, fan-in clustering was modestly but significantly more abundant in maps of propagating activity based on experimental recordings. We note that we compared thresholded graphs at the 80%-level (i.e. top 20% of non-zero edges) for this measure because the experimentally derived functional networks were not well-matched by regular lattices below this density.

Finally, we measured timing relationships among imaged active neurons. Reliable timing relationships were measured independent of other functional analyses, using cross-correlations on the normalized fluorescence traces (Methods). Presynaptic coactivity was assessed as the product of the two *z*-scored presynaptic traces and compared to postsynaptic fluorescence as a straightforward cross correlation. The resulting average cross-correlogram for fan-in triangles was stronger and more asymmetric than those measured from simple-convergence motifs (Fig 8D).

Thus, presynaptic activity in fan-in triangles was more predictive of postsynaptic firing than presynaptic activity in motifs of simple convergence. These results are consistent with fan-in triangles supporting coincident input and favoring reliable propagation of activity. Results from the model indicated that the fan-in triangle motif temporally coordinates presynaptic inputs, rendering them more capable of driving recipient neurons to threshold. Supporting our prediction of its fundamental importance for reliable recruitment, in acutely dissected neocortical tissue with more complex patterns of connectivity and intrinsic neuronal properties, we find a robust elevation of the same directed motif.

## Discussion

Using a model composed of random connections among leaky integrate-and-fire neurons with conductance-based synapses, we found that maps of propagating activity were structured and non-random. Small-world patterning in the dynamics emerged because a specific higher-order connection pattern was particularly effective for postsynaptic integration: convergence of synaptic input from connected neighbors. Synaptic connections between neighbors favored coincident timing of inputs onto their targets. This coincident activation led to efficient postsynaptic integration. As a consequence, clustering among active presynaptic cells tracked depolarization of model postsynaptic neurons. Thus, activity was preferentially routed through fan-in triangle motifs.

In experimental recordings of emergent activity in hundreds of neurons *ex vivo*, after mapping inferred recruitment patterns [33], we found that fan-in triangles were even more dramatically overrepresented than in the model. These results are contextualized by increasing recognition of non-random functional structure in networks of neurons: Rich club structure has been reported *ex vivo* and *in vivo*[31]. Clustered[30], small world functional networks[28], and nucleation of dynamics[29] have also been observed in neuronal cultures. Since cultured populations differ from neocortex in the details of their topological makeup, these findings across model systems further suggest that clustering in general and the fan-in triangle motif in particular may be a canonical feature of propagating activity among interconnected neurons. Despite differences in details of connectivity and neuronal intrinsic properties, dynamics are constrained by the requirement for coincident summation of individually weak inputs. Constraining dynamics with beyond-pairwise relationships can be helpful for cortical computation. Theoretical work has shown that non-uniform features of connection topology impact information transfer[42], and higher-order correlations were particularly impactful in low spike-rate regimes[43]. These complementary results from complex networks, statistical physics and network biology suggest that, by shaping feasible dynamics, the fan-in triangle motif could enhance information transfer from inputs to outputs.

We hypothesize that local circuits are organized around fan-in triangle motifs, promoting cooperative patterns of firing and stabilizing[44] the propagation of activity despite individually unreliable neurons. This canonical mechanism provides the coordination necessary to propagate signal despite weak synaptic connections. Indeed, reliable sequential firing was associated with number of fan-in triangles even after controlling for overall in-degree. Although clustering among fan-in triangles has not been tested directly until now, paired patch clamp recordings have shown that local neocortical circuitry is characterized structurally by abundant triplet motifs[12,39]. Our data and modeling suggest a functional consequence for a subset of these synaptic motifs: connected presynaptic neurons help establish coordinated timing among convergent inputs, leading to cooperative summation at the postsynaptic membrane. Such cooperativity has been shown to be one potential mechanism capable of generating spike trains that are consistent with experimental observations *in vivo*[45].

While there are certainly explicit developmental rules that govern neuron to neuron connectivity, our results suggest that higher-order connectivity need not require specification *a priori*. It could emerge autonomously if fan-in triangle motifs within a random network were stabilized and magnified during network development, *e*.*g*. by pruning non-recruiting connections through activity-dependent plasticity. Thus, higher-order synaptic motifs that are particularly effective for postsynaptic recruitment could potentially self-organize[46].

These results do not indicate a complete schism between synaptic connectivity and dynamics—one clearly depends on the other. However, their relationship is complicated by the integrative properties of single neurons. Synaptic integration constrains feasible dynamics, and distributed synaptic motifs route the propagation of activity. These interactions are a source of higher-order dynamical structure. The routing of information is coordinated by higher-order synaptic patterns and the context of ongoing activity because the routing of spikes is determined by relative timing and collective interactions.

## Materials and Methods

### Local cortical population model

Simulations were implemented using the Brian Brain Simulator[47]. Model populations consisted of 1000 excitatory neurons, 200 inhibitory neurons and 50 Poisson input units. Connection probabilities depended on source and target identity. For example, inhibitory-excitatory connections occurred with probability 0.25 (*P*_ee_ = 0.2, *P*_ei_ = 0.35, *P*_ie_ = 0.25, P_ii_ = 0.3).

Conductance based synaptic weights were drawn from a heavy-tailed distribution and assigned randomly[48,49]. Weights were drawn randomly from a lognormal distribution with mu = -0.64 and sigma = 0.51. These parameters are the mean and standard deviation of the corresponding normal curve. The resulting lognormal ensemble has expected mean of 0.60 and variance of 0.11, in multiples of the leak conductance. Connections from inhibitory to excitatory cells were scaled by a further 50% to simulate efficacious somatic contacts. A small tonic excitatory drive *g*_*t*_ was supplied to all units to help stabilize sparse spiking. Synaptic bombardments induced exponentially shaped membrane conductances with leaky-integrate-and-fire summation. Conductance-based synapses are important for recapitulating synaptic integration in the high-conductance state[21,50]. We used sparse and randomly connected networks in which we did not impose any synaptic organization beyond cell-type dependent connection probabilities.

Trials began with 50 ms of activity in the input pool at 15 Hz, exciting the network via random input projections. After input units were silenced, the recording period began, and activity flowed through the network for 100 ms. Input units projecting to excitatory cells randomly and independently with probability 0.1. Every 100 trials (an epoch), new random projections were drawn from the input pool to the excitatory population, simulating a diversity of activity. Participation during a single input epoch totaled 64±0.98% of neurons (mean ± std), growing to encompass 85.5% of neurons when all sets of input projections were considered (i.e. over all epochs).

Excitatory reversal potential *E*_*e*_ was 0 mV, as was *E*_*t*_. Inhibitory reversal potential *E*_*i*_ was -90 mV. Reversal potential for leak current *E*_*leak*_ was -65 mV. Firing threshold was -48 mV, and post-spike reset was -70 mV. In addition to after spike hyperpolarization induced by the reset potential, a 1 ms absolute refractory period was imposed on model neurons. Leak conductance *g*_*leak*_ was fixed at 0.20 mS. Tonic depolarizing conductance *g*_*t*_ was equal in magnitude to the leak conductance. Membrane time constant *τ*_*m*_ was 20 ms; excitatory synaptic time constant *τ*_*e*_ was 10 ms; and inhibitory synaptic time constant *τ*_*i*_ was 5 ms. Additional description can be found in[35].

### Quantification of simulated dynamics

Spiking dynamics were compared to *in vivo* activity according to the following criteria: asynchrony[51] was measured with spike-rate correlations, by convolving spike times with a Guassian kernel of width σ = 3 ms. Among excitatory neurons in the recording period, mean correlation coefficient was 0.0019[50]. This asynchrony emerged in the presence of heterogeneous connection strengths, raising the possibility of combining stable propagation with rich internal dynamics[49,52]. Irregularity was measured with interspike-intervals, which were observed to have mean squared-coefficient of variation of 0.81, consistent with other reports of irregular activity[53]. To measure inter-spike intervals, model activity was stimulated with Poisson firing for 50 ms, then allowed to evolve for 950 ms in isolation. This procedure was repeated 100 times. Excitatory spiking activity was characterized by a median branching coefficient of 1.00 (for 10 ms bins), indicating near-critical dynamics[54–57]. Firing rates in the excitatory population during the recording period were 1.33 ± 3.15 Hz (mean ± std) consistent with findings in awake behaving mice[58]. Collective spiking generated spike-driven conductances that dwarfed the leak conductance, in keeping with definitions of high-conductance state[21].

### Network construction

Call the directed network of synaptic connections among excitatory neurons E^syn^ and the population of excitatory cells V^e^. Construct the directed graph of synaptic connections:

$$G^{structural}\equiv (V^{e},E^{syn})$$

To map functional relationships using lagged firing, define *recent activity* for neuron *i* at time *t* as firing at least once in the 25 ms preceding *t*.

$$E_{ij}^{lag}\equiv P(j\;\text{active}\;|\;i\;\text{recently active})$$

$$G^{functional}\equiv (V^{e},E^{lag})$$

More formally, we can define random variable S_*i*_ representing the activity of neuron *i* such that

$$s_{i}^{t}\equiv \left\{\begin{array}{ccc} 2 & if & \text{neuron}\;i\;\text{is firing at time}\;t\\ 1 & if & \text{neuron}\;i\;\text{is not firing but was active within the last}\;25\;\text{ms}\\ 0 & otherwise & \end{array}\right\}$$

In that case,

$$E_{ij}^{lag}\equiv P(s_{j}=2\;|\;s_{i}>0)$$

The recruitment network encompassed synaptically connected neurons manifesting lagged patterns:

$$E_{ij}^{omniscient}\equiv \left\{\begin{array}{ccc} E_{ij}^{lag} & if & E_{ij}^{syn}>0\\ 0 & otherwise & \end{array}\right\}$$

$$G^{recruitment}\equiv (V^{e},E^{omniscient})$$

Iterative Bayesian networks were measured with a heuristic optimization procedure, described further below and in [35], following [28].

### Global network statistics

Since shortest path measurements assume a cost matrix, edge weights were first inverted so strong connections were cheap and zero-weighted connections were infinitely costly. Shortest paths between all pairs were computed using Dijkstra’s algorithm. Mean path length was compared to sparseness-matched Erdős-Rényi graphs analyzed in the same way. Local clustering coefficients were computed using the neighbors of neighbors formulation[32] and aggregated as the mean over all neurons. Sparseness-matched Erdős-Rényi graphs were analyzed in the same fashion. Clustering score was the ratio of the actual mean to sparseness-matched null mean. Small-world topologies can be quantified as a ratio of ratios, clustering elevation divided by mean path length reduction[33].

### Transitive clustering and directed clustering

Clustering was also investigated using a related definition, the number of connected undirected triangles as a fraction of all possible undirected triangles (transitivity formulation). Directed clustering was computed in the same way, using directed triangles instead of undirected[38].

To compare clustering between data and model networks, across connection densities that were very different, we followed the small-world propensity approach[41]. In that work, clustering levels ΔC are normalized as the fractional distance between density-matched lattice and random graphs. We termed this measure clustering propensity, expressing it as 1 – ΔC so that 1 signified extreme clustering and 0 signified no clustering beyond that expected at random. We made a straightforward extension to this approach to account for directed clustering, simply substituting directed triangle counts for undirected triangle counts, with appropriate normalizations[38]. Quantifications based on clustering propensity recapitulated our findings quantifying clustering as fractional abundance over random expectation.

### Mapping presynaptic ensemble in relation to postsynaptic voltage

For the set of voltage bins with lower bounds **a** and upper bounds **b**, construct one network for each bin *k*, where edge (*i*, *j*)^*k*^ is quantifying the probability model neuron *j* will have postsynaptic potential M_j_ between **a**_k_ and **b**_k_ conditioned on presynaptic model neuron *i* being recently active. *Recently active* was defined as firing within 25 ms relative to postsynaptic voltage measurement. A final condition was imposed: that connected pairs also share a synaptic connection, a convenience of measurement unique to simulated networks.

$$G^{k}\equiv (V^{e},E^{k})$$

$$E_{j}^{k}\equiv \left\{\begin{array}{ccc} P(a_{k}<M_{j}<b_{k}\;|\;i\;\text{recently active}) & if & E_{ij}^{syn}>0\\ 0 & otherwise & \end{array}\right\}$$

### Scaled synaptic topologies

Functional topologies were measured for simulations having typical synaptic weight distributions (n = 5) and for simulations where random draws from the synaptic weight distributions were scaled to double strength (n = 6). Ratios for global clustering, characteristic path, and smallworldness were quantified following[33], as above, on the two sets of weighted, symmetrized topologies. Directed clustering was measured following[38]. The directed clustering measurements were conducted on binary topologies to control for potential differences stemming from their different underlying mean synaptic weights.

### Preparation of Ca^2+^-dye loaded slices

All procedures were performed in accordance with and approved by the Institutional Animal Care and Use Committee at the University of Chicago. One juvenile mouse (postnatal day 14, of strain C57BL/6) was anesthetized by intraperitoneal injection of ketamine-xylazine and rapidly decapitated. The brain was dissected and placed in oxygenated, ice-cold artificial cerebrospinal fluid (Cut-ACSF; contents contain the following in mM: 3 KCl, 26 NaHCO3, 1 NaH2PO4, 0.5 CaCl2, 3.5 MgSO4 25 dextrose, and 123 sucrose). The brain was then sliced coronally using a vibratome (VT1000S; Leica) into 450 μm thick slices. These slices encompassed the mouse whisker somatosensory cortex. Slices were then transferred into 35°C oxygenated incubation fluid (Incu-ACSF; contents contain the following, in mM: 123 NaCl, 3 KCl, 26 NaHCO3, 1 NaH2PO4, 2 CaCl2, 6 MgSO4, 25 dextrose) for 30 min. Bulk loading of Ca^2+^ dye was then performed, via transfer of slices into a Petri dish containing ∼2 ml of Incu-ACSF and an aliquot of 50 μg Fura-2AM (Product code, Invitrogen, location) dissolved in 13 μl DMSO and 2 μl of Pluronic F-127 (Code, Invitrogen, location) (following [9]).

### Ca^2+^-imaging procedure

Throughout the duration of imaging, slices were continuously perfused with a standard ACSF solution (contents contain the following, in mM: 123 NaCl, 3 KCl, 26 NaHCO_3_, 1 NaH_2_PO_4_, 2 CaCl_2_, 2 MgSO_4_, and 25 dextrose, which was continuously aerated with 95% O_2_, 5% CO_2_). Visualization of Fura-2AM loaded neurons was performed via serial 5 min recordings, collected using the HOPS scanning technique (a suite of software and custom microscopy setup developed in-house, see [40]). This method allowed us to monitor action potential generation within individual neurons, by detecting contours of loaded cells from a raster image, then computing an efficient traveling salesman tour over those cell bodies. Our dwell time parameter was fixed at a value of 16 samples/cell/frame. Population framerate was 20 Hz, resulting in ~450 neurons sampled once every ~50 ms. Changes in emitted fluorescence were analyzed with a threshold-crossing approach. First, a signal-to-noise cutoff was implemented by measuring the ratio of the 99^th^ percentile divided by the mean for the fluorescence trace of each cell. Cells exceeding 1.55 by this metric were retained for further analysis. Of the 444 sampled neurons, 189 exceeded our strict criterion on signal-to-noise (see Methods). Among these cells with clean fluorescent signals, instances of elevated firing were identified from excursions in the signal exceeding two-sigma, with inflection points more precisely identified by following these excursions backwards to the bin of their most recent median-crossing. The resulting binary vector identified high-probability periods of spiking activity across the imaged population[14,59].

### Inferring connectivity

Recurring timing relationships can be used to identify likely synaptic connections between individual pairs, particularly lagged firing near the timescale of synaptic integration. We used an iterative Bayesian inference algorithm to parse these lagged firing patterns[28,35]. The inference algorithm was initialized five times, and final weights were pooled as an average. The combined network was thresholded to isolate its strongest relationships. With increasing threshold, functional relationships became more precise in indicating true monosynaptic connectivity, and also more confidently overabundant in the fan-in triangle motif.

To understand the impact of mistaken inferences from a different perspective, independent of relationships between functional weight and true connectivity, bootstrap resampling was used to estimate how errors in inferred connectivity affected estimates of directed clustering measures. For an error rate of 30% estimated from simulated experimental constraints[35], differences in directed clustering were significant even after redacting possible false positives (100 bootstrap-resampled topologies; Fig 8C and 8E).

In a typical simulated network, the density of the recruitment network was 0.049, meaning only about one quarter of synaptic connections were a site of propagating activity. Since only those pairs are visible in patterns of lagged firing, the density of recruiting connections was shown for an additional definition of optimal performance (one potentially more appropriate for models with sparse firing).

### Validating inferred relationships with cross-correlation

Average cross-correlations were computed over a two-second sliding window using *z*-scored fluorescence traces. The first signal was computed as the product of two putative presynaptic fluorescence traces, as a simple score of their activity and/or coactivity. The second signal was the postsynaptic fluorescence trace. Their raw cross-correlation measures the timing offsets between putative presynaptic activity and postsynaptic firing. The functional relationships used to define fan-in triangle motifs versus simple convergence motifs inferred using iterative Bayesian inference, on the basis of single-frame lagged activity, measured in 50 ms bins.
